# The fate of extra centrosomes in newly formed tetraploid cells: should I stay, or should I go?

**DOI:** 10.3389/fcell.2023.1210983

**Published:** 2023-07-27

**Authors:** Mathew Bloomfield, Daniela Cimini

**Affiliations:** Department of Biological Sciences and Fralin Life Sciences Institute, Virginia Tech, Blacksburg, VA, United States

**Keywords:** tetraploidy, cancer evolution, centrosomes, whole genome doubling, genomic instability, centrosome amplification

## Abstract

An increase in centrosome number is commonly observed in cancer cells, but the role centrosome amplification plays along with how and when it occurs during cancer development is unclear. One mechanism for generating cancer cells with extra centrosomes is whole genome doubling (WGD), an event that occurs in over 30% of human cancers and is associated with poor survival. Newly formed tetraploid cells can acquire extra centrosomes during WGD, and a generally accepted model proposes that centrosome amplification in tetraploid cells promotes cancer progression by generating aneuploidy and chromosomal instability. Recent findings, however, indicate that newly formed tetraploid cells *in vitro* lose their extra centrosomes to prevent multipolar cell divisions. Rather than persistent centrosome amplification, this evidence raises the possibility that it may be advantageous for tetraploid cells to initially restore centrosome number homeostasis and for a fraction of the population to reacquire additional centrosomes in the later stages of cancer evolution. In this review, we explore the different evolutionary paths available to newly formed tetraploid cells, their effects on centrosome and chromosome number distribution in daughter cells, and their probabilities of long-term survival. We then discuss the mechanisms that may alter centrosome and chromosome numbers in tetraploid cells and their relevance to cancer progression following WGD.

## 1 Introduction

The centrosome is a small cytoplasmic organelle responsible for microtubule nucleation and organization. It also acts as a signaling hub for proteins involved in multiple cellular processes, including cell adhesion, DNA damage repair, and cell cycle progression (Conduit et al., 2015; Mullee and Morrison, 2016). Regulation of centrosome number is critical for cell health, and defects in both centrosome number and structure contribute to disease (Badano et al., 2005; Bettencourt-Dias et al., 2011; Goundiam and Basto, 2021). Centrosome duplication, a key event in the centrosome cycle, is coupled to DNA replication and occurs only once during a typical cell cycle. Cells should begin G1 with a single centrosome consisting of a mother and a daughter centriole, which are duplicated in S phase to generate two centrosomes that will form the poles of the mitotic spindle. In mitosis, the centrosomes are distributed between the two daughter cells, producing two new G1 cells with a single centrosome (Nigg and Holland, 2018). Centrosome duplication is controlled by a group of core proteins that are often expressed at different levels in cancer cells, which can disrupt centrosome homeostasis (Fukasawa, 2007; Chan, 2011; Nigg and Stearns, 2011; Nigg and Holland, 2018). Centrosome amplification, or cells that have a higher than normal number of centrioles, is common in human cancers and is associated with invasion, metastasis, and poor clinical outcome (Chan, 2011; LoMastro and Holland, 2019). This has prompted investigators to develop drugs that target various centrosome functions as a therapeutic strategy against cancers with centrosome amplification (reviewed in (Godinho et al., 2009; Godinho and Pellman, 2014; Cosenza and Krämer, 2016).

Supernumerary centrosomes can also arise through whole genome doubling (WGD) events, such as cell fusion, mitotic slippage, or cytokinesis failure (Ganem et al., 2007; Godinho and Pellman, 2014; Lim and Ganem, 2014). As a result of WGD, newly formed tetraploid cells typically have twice the number of chromosomes and centrosomes compared to diploid cells (Ganem et al., 2009; Baudoin et al., 2020). WGD is a frequent event in cancer evolution (Carter et al., 2012; Zack et al., 2013; Dewhurst et al., 2014; Bielski et al., 2018; Taylor et al., 2018; López et al., 2020; Cohen-Sharir et al., 2021; Quinton et al., 2021; Prasad et al., 2022), suggesting that WGD may represent a common route to centrosome amplification in human cancers. A popular theory is that the extra centrosomes acquired during WGD are important for cancer progression because they promote the accumulation of aneuploidy and chromosomal instability (CIN) in proliferating tetraploid cells (Ganem et al., 2007; Godinho et al., 2009; Zhang and Pellman, 2022). In cell culture models, however, newly formed tetraploid cells tend to lose extra centrosomes within a few cell cycles following WGD while maintaining near-tetraploid genomes as they proliferate (Baudoin et al., 2020). These paradoxical observations suggest that our knowledge about the fate of the extra centrosomes after WGD in cancers is incomplete, particularly regarding the evolution of newly formed tetraploid cells *in vivo*. There are a few possibilities that may explain this discrepancy: extra centrosomes can be lost in WGD+ cells; alternatively, they can be preserved by centrosome clustering or inactivation mechanisms that prevent catastrophic multipolar cell divisions; finally, cells that initially lose extra centrosomes after WGD may increase centrosome numbers later through cell-intrinsic events and/or environmental factors within the tissue/tumor microenvironment (TME). Furthermore, WGD can promote genome instability and increase the tumorigenicity of cell lines regardless of whether the extra centrosomes are preserved or not (Fujiwara et al., 2005; Dewhurst et al., 2014; Kuznetsova et al., 2015; Prasad et al., 2022). In this review, we describe how extra centrosomes can be distributed in the early mitoses that follow WGD, how they can be preserved in newly formed tetraploid cells via centrosome clustering, and how they can be reacquired in WGD+ cells with normal centrosome numbers due to overduplication. Finally, we discuss how WGD leads to genomic instability in normal and cancer cells.

## 2 Centrosome and chromosome partitioning during the evolution of newly formed tetraploid cells

**FIGURE 1:**
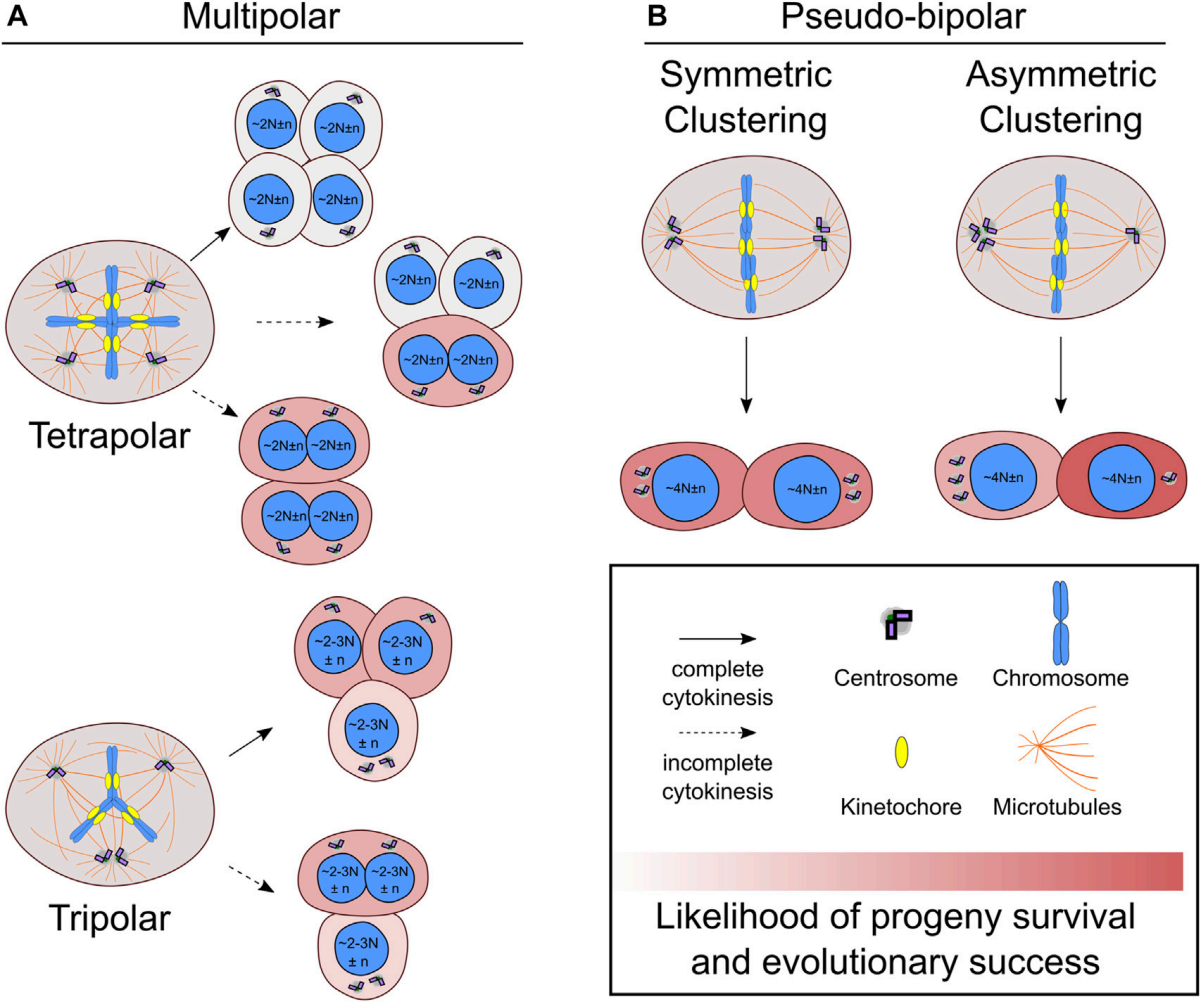
Centrosome and chromosome partitioning in the first mitosis after whole genome doubling. **(A)** Newly formed tetraploid cells that enter mitosis with four centrosomes may undergo multipolar cell divisions, which can be tetrapolar (**A**, top) or tripolar (**A**, bottom). Incomplete cytokinesis (dotted line) may occur during multipolar divisions, which will generate fewer daughters than would be expected based on the number of anaphase spindle poles. **(B)** Newly formed tetraploid cells can also cluster the extra centrosomes symmetrically (**B**, left) or asymmetrically (**B**, right) to form a pseudo-bipolar spindle. The daughter cell that inherits a single centrosome during asymmetric centrosome clustering will no longer be at risk of undergoing multipolar cell divisions and has the highest likelihood of long-term evolutionary success compared to any other outcome. N indicates basal ploidy level of nucleus, and, in some outcomes, the resulting ploidy may be between 2N and 3N (e.g., hyper-diploid or hypo-triploid). Chromosome number variation around basal ploidy is denoted by n.

Newly formed tetraploid cells that enter G1 with two centrosomes will have twice as many in the next mitosis if both centrosomes are replicated once. Tetraploid cells attempting to divide with four centrosomes often form mitotic spindles with more than two poles that can unevenly distribute chromosomes and centrosomes to the daughter cells (Ganem et al., 2009; Baudoin et al., 2020). Multipolar cell divisions tend to be catastrophic events with a high risk of cell death or arrest (Ganem et al., 2009; Baudoin et al., 2020), but there are several possible outcomes that affect centrosome and chromosome numbers differently. One outcome is a tetrapolar cell division resulting in four daughter cells with a single centrosome (Figure 1A). Even though normal centrosome number is restored, the daughter cells formed by tetrapolar divisions are likely to inherit karyotypes with several chromosomal monosomies or even nullisomies, which can explain the high rates of cell death in the progeny of multipolar divisions (Vitale et al., 2010; Baudoin et al., 2020).

It is also possible for varying degrees of centrosome clustering to occur in newly formed tetraploid cells undergoing mitosis with four centrosomes (Figure 1). In tripolar spindles, two centrosomes cluster together to form a single spindle pole, while the other two centrosomes each form their own spindle pole. This will restore centrosome homeostasis in two of the daughter cells. The other daughter cell will still have two centrosomes and risk undergoing another multipolar division upon reentering mitosis. Tripolar cell divisions also generate highly aneuploid daughter cells prone to cell arrest or death (Baudoin et al., 2020). During multipolar divisions, it is also possible for cytokinesis to fail along one of the division planes, resulting in fewer daughter cells than anaphase spindle poles (Wheatley and Wang, 1996; Chen et al., 2016; Baudoin et al., 2020) (Figure 1A). In these cases, there will also be asymmetric distribution of centrosomes and chromosomes to the daughter cells. Since the chromosomes are partitioned to fewer cells, it may be more likely for multipolar divisions with fewer daughter cells to produce viable progeny compared to multipolar divisions with complete cytokinesis. Such outcomes could result in hyper-tetraploid and hypo-tetraploid daughter cells with extra centrosomes. The hyper-tetraploid cell would be again prone to multipolar division, but having more chromosomes may increase the likelihood of yielding daughter cells with viable yet highly aneuploid karyotypes. Although centrosome fate was not tracked, such an outcome was observed in polyploid HeLa cells, which have more chromosomes than newly formed tetraploid cells (Chen et al., 2016). Polyploid HeLa cells undergoing multipolar mitosis followed by incomplete cytokinesis, yielded two daughter cells that tended to be viable and proliferative (Chen et al., 2016). Multipolar divisions, though often unsuccessful, can generate aneuploid cells with normal or extra centrosomes. One study showed that daughter cells of lower ploidy isolated after multipolar divisions were capable of forming tumors in mice (Vitale et al., 2010). However, it is unclear to what extent multipolar divisions after WGD may contribute to punctuated genomic evolution—a saltational jump from tetraploid to highly aneuploid karyotypes with hyper-diploid or near-triploid chromosome numbers—in human cancers.

Newly formed tetraploid cells can also effectively cluster their extra centrosomes to assemble a spindle with two poles and undergo a bipolar cell division (Baudoin et al., 2020). Compared to multipolar cell divisions, this outcome is less severe and is more likely to produce viable daughter cells with near-tetraploid chromosome numbers. The fate of the daughter cells, however, varies depending on how the centrosomes are distributed (Figure 1B). In symmetric centrosome clustering, the two spindle poles each have a pair of centrosomes, meaning that each daughter cell will have abnormal centrosome numbers and may undergo a multipolar division in the next mitosis. Alternatively, in asymmetric clustering, three centrosomes cluster at one spindle pole, while the other spindle pole has a single centrosome. This outcome would allow the daughter cell with normal centrosome numbers to proliferate without substantial risk of spindle multipolarity in the following mitoses and may favor tetraploid cell evolution (Baudoin et al., 2020).

Effective centrosome clustering mechanisms would provide a selective advantage for newly formed tetraploid cells and, more generally, all cancer cells with supernumerary centrosomes (Brinkley, 2001; Nigg, 2002; 2006). Some centrosome clustering mechanisms that have been characterized in newly formed tetraploid (or polyploid) cells involve mitotic proteins that function in spindle formation and the spindle assembly checkpoint (SAC), although several other factors are also likely to contribute [for a more comprehensive review on centrosome clustering mechanisms, please see (Godinho et al., 2009; Krämer et al., 2011; Godinho and Pellman, 2014)]. The kinesin HSET (KIFC1), a minus end-directed microtubule motor protein that cross-links and slides microtubules to help focus spindle poles, plays an important role in centrosome clustering (Kwon et al., 2008; Vitre et al., 2020). Increasing HSET expression reduced multipolar divisions in polyploid HeLa cells (Chen et al., 2016). One study found that HSET expression was higher in WGD+ compared to WGD− prostate cancers, but it is unclear if these samples also had high levels of cancer cells with supernumerary centrosomes (Kostecka et al., 2021). Stabilizing spindle microtubules by depleting MCAK (KIF2C), a kinesin that removes tubulin from microtubule minus-ends, promoted centrosome clustering in the early stages of mitosis and allowed polyploid cells to form bipolar spindles with extra centrosomes (Goupil et al., 2020). It is possible that longer astral microtubules improve the efficiency of HSET-mediated clustering of adjacent centrosomes. Low levels of active Eg5, a mitotic kinesin that slides antiparallel microtubules apart to separate the spindle poles and opposes the inward pulling forces of HSET (Mountain et al., 1999), also increased the fraction of bipolar cell divisions in polyploid HeLa cells (Shu et al., 2019). Consistently, loss of the anaphase-promoting complex/cyclosome (APC/C), an E3 ligase that regulates mitotic progression by marking specific proteins for degradation, increased Eg5 levels in the spindle and decreased centrosome clustering efficiency in diploid and tetraploid DLD-1 cells with extra centrosomes (Drosopoulos et al., 2014). Centrosome clustering efficiency in newly formed tetraploid cells improved after depletion of the deubiquitinating enzyme USP28, which interacted with NuMa—a protein that bundles spindle microtubules to help focus and position spindle poles—at centrosomes in tetraploid cells (Bernhard et al., 2022). High levels of NuMa, which organizes the minus ends of spindle microtubules, can displace dynein, a motor protein important for spindle positioning, and lead to spindle multipolarity in cells with extra centrosomes (Quintyne et al., 2005), suggesting that low levels of USP28 may promote centrosome clustering by increasing the proteasomal degradation of NuMa during or prior to spindle assembly. These findings show that altering the expression of key mitotic proteins can change the balance of spindle forces in a manner that favors centrosome clustering in newly formed tetraploid cells.

A functional SAC, a signaling pathway that prevents anaphase onset until kinetochores have become attached to spindle microtubules (Musacchio, 2015), may give newly formed tetraploid cells more time to cluster their extra centrosomes. Consistently, mitosis often takes longer in cells with extra centrosomes because of difficulties in satisfying the SAC (Yang et al., 2008). Recent findings also suggest that inefficient SAC silencing, perhaps by also lengthening mitosis to allow for centrosome clustering, may provide a selective advantage for newly formed tetraploid cells. The regulatory subunits of PP2A, a serine/threonine phosphatase that removes phosphorylation marks to silence SAC signaling during mitosis (Espert et al., 2014), are frequently mutated in WGD+ human cancers (Zack et al., 2013; Quinton et al., 2021). One study showed that *PPP2R1A* missense mutations perturbed PP2A function by disrupting interactions with its B55 and B56 subunits and that, although the mechanism was not identified, PP2A inactivation promoted centrosome clustering in newly formed tetraploid cells (Antao et al., 2019). Nevertheless, it is unclear if WGD+ cancers with PP2A inactivating mutations have a higher fraction of cells with supernumerary centrosomes *in vivo* than WGD+ cancers with functional PP2A.

Although clustering extra centrosomes to undergo bipolar cell divisions can protect daughter cells from high levels of aneuploidy and potential death, it may still lead to increased levels of chromosome missegregation compared to cells with normal centrosome numbers. In cells with extra centrosomes, the spindle may initially assemble with multiple poles that cluster during mitotic progression (Ganem et al., 2009; Silkworth et al., 2009). Transient spindle multipolarity increases the risk of kinetochores binding to microtubules from opposing spindle poles (i.e., merotelic attachment). If a merotelic attachment persists into anaphase, the chromosome is pulled in opposite directions and can lag in the spindle midzone rather than segregating with the other chromosomes (Cimini et al., 2001). Lagging chromosomes that do not segregate to the correct daughter cell and/or do not get incorporated into the main nucleus lead to whole-chromosome or segmental aneuploidy (Cimini, 2008). Lagging chromosomes that are separated from the main nucleus in a micronucleus typically are prone to further missegregation in the next mitosis (He et al., 2019). Micronuclei may also assemble defective nuclear envelopes that are unable to transport proteins necessary for DNA replication and can rupture, exposing the missegregated chromosome(s) to cytosolic nucleases. As a result, chromosomes trapped in micronuclei are prone to incomplete replication, DNA breaks, and chromosomal shattering leading to complex rearrangements (i.e., chromothripsis) (Crasta et al., 2012; Hatch et al., 2013; Zhang et al., 2015; Liu et al., 2018; Tang et al., 2022). There is evidence micronucleus instability can cause punctuated bursts of numerical and structural chromosomal alterations within a few cell divisions and promote inflammation, epithelial-to-mesenchymal transition, and metastasis (Mackenzie et al., 2017; Bakhoum et al., 2018; Umbreit et al., 2020), but we are still learning about the cellular events leading to micronuclei rupture in normal and cancer cells (Sepaniac et al., 2021; Agustinus et al., 2022; Mammel et al., 2022; Papathanasiou et al., 2022). Chromothripsis is more frequent in WGD+ than WGD− cancers (Notta et al., 2016; Cortés-Ciriano et al., 2020), likely reflecting an increase in both the rates and tolerance of genomic alterations in tetraploid cells. Because these events are often clonal and occur early in cancer development, it is tempting to speculate that chromothripsis may happen shortly after WGD and could be common in newly formed tetraploid cells dividing with extra centrosomes.

Overall, there appear to be two main options for the long-term evolutionary success of newly formed tetraploid cells: losing extra centrosomes via asymmetric clustering in bipolar spindles or retaining extra centrosomes and avoiding multipolar cell divisions via symmetric clustering in bipolar spindles. Even if the first cell division following WGD is bipolar, there is still a fair probability that asymmetric clustering may not occur and the daughter cells will still have four centrosomes and near-tetraploid genomes in the next mitosis (Baudoin et al., 2020). The high number of chromosomes and centrosomes can complicate centrosome clustering, leaving these tetraploid cells at risk of undergoing lethal multipolar cell divisions. For instance, chromosomes can form physical barriers between spindle poles (Goupil et al., 2020), so there will be more steric impediments to centrosome clustering in cells with tetraploid compared to diploid genomes. Similarly, bipolar spindle formation becomes more difficult as centrosome number increases. Even cancer cell lines with high levels of centrosome amplification that are adept at clustering extra centrosomes (e.g., N1E-115, MDA-MB-231, Caco2) still experience multipolar divisions with some regularity (Ganem et al., 2009). This suggests that restoring centrosome homeostasis would be beneficial for the early expansion of newly formed tetraploid cells in developing cancers. As the tetraploid cell population expands and cell death becomes less costly, it may then be functionally valuable for centrosome amplification to also increase and propagate genomic instability.

## 3 Cellular and environmental factors can disrupt centrosome number homeostasis

**FIGURE 2:**
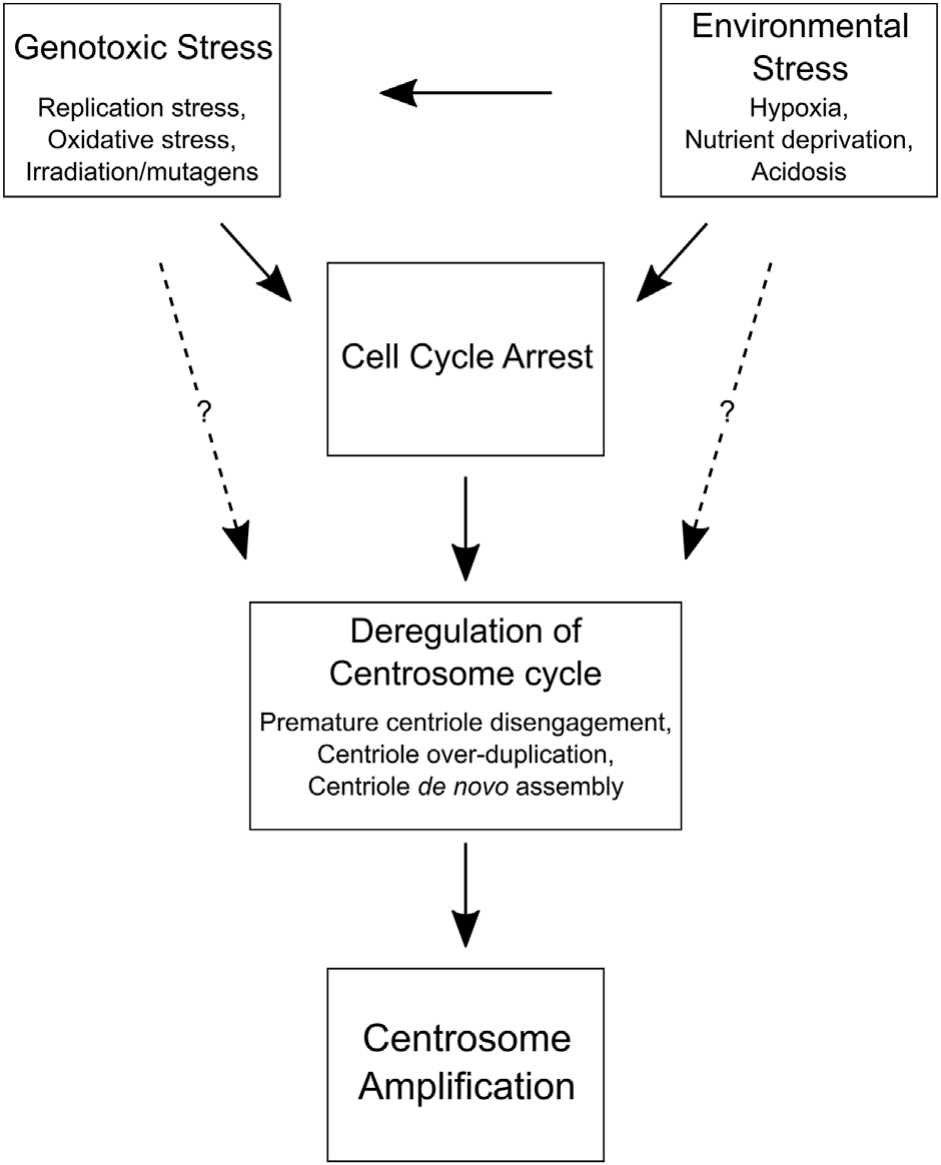
Cell and environmental factors that promote centrosome amplification. A flow chart depicting how genotoxic and environmental stress can lead to centrosome amplification by inducing a cell cycle delay. Prolonged interphase can cause centrosome cycle deregulation and centrosome amplification in normal and cancer cells. Although the mechanisms are unclear (dotted lines), it may also be possible for genotoxic and environmental stress to directly disrupt the centrosome cycle and promote centrosome amplification without a cell cycle delay, perhaps by altering the expression of key centrosome replication factors (e.g., PLK1, PLK4, etc.) or DNA damage response proteins (e.g., CHK1, CHK2, ATM, ATR, etc.).

There is a growing list of cellular and environmental factors that promote centrosome amplification in normal and cancer cells. WGD is an example of centrosome accumulation, where a cell inherits multiple centrosomes that were properly duplicated (Krämer et al., 2011). This is an effective mechanism for generating cells with extra centrosomes, but, as described earlier, multipolar cell divisions are frequent after WGD and can lead to cell death (Ganem et al., 2009; Baudoin et al., 2020). Even if the extra centrosomes acquired via WGD are initially lost, tetraploid cells could reacquire abnormal centrosome numbers later through other mechanisms (Figure 2). Indeed, cells can increase centrosome number without doubling their genome via centriole over-duplication and *de novo* centriole assembly (Khodjakov et al., 2002; Habedanck et al., 2005; La Terra et al., 2005; Kleylein-Sohn et al., 2007; Wang W. J. et al., 2015; Denu et al., 2018). Cells that inherit an abnormal number of duplication-competent daughter centrioles can enter mitosis with more than two centrosomes and risk undergoing multipolar divisions unless centrosome clustering occurs, much like the mitotic events observed in newly formed tetraploid cells (Figure 1).

Centrosome amplification can result from deregulation of centrosome duplication (for a comprehensive review of normal centrosome duplication, please see (Mardin and Schiebel, 2012)), which is governed by a relatively small number of core proteins (Nigg and Stearns, 2011) that are often aberrantly expressed in human cancers (Starita et al., 2004; Fukasawa, 2007; Loncarek et al., 2008; Chan, 2011; Nigg and Holland, 2018). Overexpression of Polo-like kinase 4 (PLK4), the master regulator of centrosome duplication, can lead to centriole overduplication and generate cells with extra centrosomes without increasing ploidy (Habedanck et al., 2005; Kleylein-Sohn et al., 2007; Serçin et al., 2016; Levine et al., 2017). High levels of PLK4 were also capable of inducing *de novo* centriole assembly and maturation in flies (Nabais et al., 2021). PLK4 abundance is primarily controlled by proteolysis, so altered expression of its regulators (e.g., βTrCP) may promote PLK4 accumulation and centrosome amplification (Cunha-Ferreira et al., 2009; Rogers et al., 2009; Holland et al., 2010). PLK4 levels were shown to be regulated by p53, which suppresses PLK4 expression in conditions of physiological stress (Li et al., 2005; Nakamura et al., 2013). p53 can also suppress centrosome amplification via its effect on cell cycle progression. p53 is activated by the PIDDosome, a protein complex containing PIDD1, RAIDD, and Caspase-2 (Tinel and Tschopp, 2004), to induce a cell cycle arrest when extra mother centrioles are detected (Fava et al., 2017). Thus, although p53 loss *per se* does not directly lead to centrosome amplification (Fukasawa et al., 1996; Marthiens et al., 2013), p53 function is commonly inactivated prior to WGD during cancer evolution (Carter et al., 2012; Zack et al., 2013; Bielski et al., 2018) and would be permissive for the proliferation of newly formed tetraploid cells or cancer cells with extra centrosomes.

Abnormal centrosome numbers in normal and cancer cells have been reported following DNA replication stress, oxidative stress, irradiation, and exposure to mutagenic drugs (Balczon et al., 1995; Sato et al., 2000; Meraldi et al., 2002; Chae et al., 2005; Kawamura et al., 2006; Prosser et al., 2009; Saladino et al., 2009; Douthwright and Sluder, 2014). However, what causes centrosome amplification after genotoxic stress is still unclear and may depend on multiple factors, such as the cell’s genetic background, the source and type of DNA damage, and when in the cell cycle DNA damage occurs (Saladino et al., 2012; Mullee and Morrison, 2016). Several components of the DNA damage response (DDR) are found at centrosomes and interact with regulators of centrosome duplication (Brown et al., 1994; Krämer et al., 2004; Oricchio et al., 2006; Zhang et al., 2007), while some centrosome components also have known roles in DNA repair (Nishi et al., 2005; Sivasubramaniam et al., 2008; Pan and Lee, 2009; Dantas et al., 2011). This raises the possibility that centrosome amplification is a programmed response to genotoxic stress mediated by signaling between DDR and centrosome proteins (Mullee and Morrison, 2016). In support of this theory, the DDR kinases ATM, CHK1, and CHK2 all contribute to DNA damage-induced centrosome amplification (Dodson et al., 2004; Bourke et al., 2007; Bourke et al., 2010; Wang C. et al., 2015). Centrosome amplification following genotoxic stress could also be an indirect consequence of DDR signaling, which may increase the likelihood of centriole duplication errors by arresting the cell cycle. Centriole overduplication and *de novo* centriole assembly are both observed in response to prolonged interphase (Balczon et al., 1995; Khodjakov et al., 2002; Stucke et al., 2002; Lončarek et al., 2010). Genotoxic stress is also associated with centriole splitting and structural damage to centrosomes, which can also lead to centrosome amplification (Saladino et al., 2009; Inanç et al., 2010; Antonczak et al., 2016). One common player in many of these processes is PLK1, which has important roles in cell cycle progression and the centrosome cycle (Shukla et al., 2015; Gheghiani et al., 2017). PLK1 is a target of the DDR kinases ATM and ATR (Smits et al., 2000; van Vugt et al., 2001) as well as cyclin-dependent kinases (e.g., CDK1 and CDK2) (van Vugt et al., 2010; Wakida et al., 2017), which are regulated by CHK1 and CHK2 following DNA damage (Zannini et al., 2014; Smits and Gillespie, 2015). While it is clear that genotoxic stress can lead to changes in centrosome number, further work is needed to fully elucidate the pathway(s) regulating this response and whether they play a role in promoting centrosome amplification in human cancers.

There is also evidence that centrosome amplification can result from changes in the cellular and physical composition of the TME. In most cases, tumors do not have the vasculature required to support waste removal and nutrient supply throughout the tumor tissue (Gillies et al., 2018). This can create environmental conditions, such as hypoxia, acidosis, nutrient scarcity, and inflammation, that may not exist in normal tissues and have biological effects on cancer cells (Alfarouk et al., 2013). Several studies have shown that hypoxia, or oxygen deficiency, promotes centrosome amplification (Nakada et al., 2011; Mittal et al., 2020; Mittal et al., 2022). One study found a positive correlation between hypoxia- and centrosome amplification-related gene expression in human cancers (de Almeida et al., 2019). HIF1α and PLK4 expression were also correlated in clinical samples, suggesting that hypoxia-induced centrosome amplification may be driven by HIF1α-dependent upregulation of PLK4 (Mittal et al., 2022). However, there is also evidence that hypoxia may lead to excessive accumulation of Cep192, a component of the pericentriolar wall, and prevent centrosome duplication, resulting in mitotic cells with only two centrioles (Moser et al., 2013). Whether this pathway is circumvented or inactivated to enable hypoxia-induced centrosome amplification in cancer cells remains unclear. Insufficient glucose availability and lactic acidosis, which can result from poor tumor vasculature and tend to co-occur with hypoxia, also increased centrosome abnormalities and spindle multipolarity in a p53-null breast cancer cell line (Dai et al., 2013). Heat stress, a consequence of high temperatures in the TME, is also linked to centrosome amplification (Vidair et al., 1993; Nakahata et al., 2002; Gupta and Srinivas, 2008; Petrova et al., 2016; Baek et al., 2017), due to centriole overduplication (Petrova et al., 2016) or *de novo* centriole assembly (Baek et al., 2017). In many cases, the mechanism(s) for how stressful environmental conditions, such as the ones described above, cause centrosome amplification *in vivo* has not been determined. One interesting possibility is that environmental stress perturbs centrosome duplication in cancer cells by increasing the intracellular levels of reactive oxygen species (ROS), inducing DNA damage, and/or delaying cell cycle progression. These factors are all associated with centrosome amplification in cell culture models, but further investigation is required to determine whether they contribute to centrosome amplification in cancers. It will also be important to examine the effect of stromal cells in the TME, which engage in a bidirectional interplay with the local environment (Merlo et al., 2006; Ibrahim-Hashim et al., 2017), on centrosome homeostasis in cancer cells.

## 4 The functional (non-mitotic) consequences of having extra centrosomes

There is accumulating evidence that harboring an excess number of centrosomes has functional consequences for normal and cancer cells during interphase. Tetraploid MCF10A mammary epithelial cells with extra centrosomes were more invasive in a three-dimensional (3D) cell culture system compared to tetraploid MCF10A cells that had lost their extra centrosomes (Godinho et al., 2014). This invasive phenotype in the mammary cells with supernumerary centrosomes was due to elevated microtubule nucleation that induced Rac1 activity, which in turn increased actin polymerization, decreased cell-to-cell adhesion, and promoted extracellular matrix (ECM) degradation (Godinho et al., 2014). Similarly, centrosome amplification in *Drosophila* tracheal cells led to altered cytoskeletal structure and increased single-cell branching *in vivo*, a process that is critical for lumen formation and resembles blood vessel sprouting during angiogenesis (Ricolo et al., 2016). These findings demonstrate that cells with extra centrosomes display altered cytoskeletal organization and dynamics, which contribute to the formation of invasive cellular structures in flies and 3D cell culture models.

Cells with extra centrosomes can also affect the behavior of adjacent cells through paracrine signaling (Arnandis et al., 2018; Adams et al., 2021). The invasiveness of MCF10A cells in 3D cultures and zebrafish increased when exposed to conditioned media from cells with extra centrosomes driven by PLK4 overexpression compared to conditioned media from control cells without supernumerary centrosomes (Arnandis et al., 2018). Non-cell-autonomous invasion triggered by centrosome amplification generated protrusions containing nuclei, indicative of collective migration, which was distinct from the cell-autonomous invasion observed in cells with extra centrosomes (Godinho et al., 2014; Arnandis et al., 2018). Cells with extra centrosomes also displayed a secretory phenotype—similar to senescent cells (Coppé et al., 2010)—that was caused by ROS-induced oxidative stress (Arnandis et al., 2018). This led to the secretion of interleukin-8 (IL-8), a pro-inflammatory chemokine associated with cancer cell invasion (Waugh and Wilson, 2008), which increased the invasiveness of neighboring cells through the transactivation of HER2 (Arnandis et al., 2018). Similarly, centrosome amplification, IL-8 secretion, and cellular invasion were correlated in subclones derived from breast cancer cell lines (Liu et al., 2015). Finally, pancreatic cancer cell lines with centrosome amplification had impaired lysosomal function from excessive ROS production, which promoted the secretion of small extracellular vesicles (Adams et al., 2021). These changes in exosome secretion may be connected to the disruption of autophagy, which is induced by oxidative stress and influences the distribution of exosomes (Filomeni et al., 2015; Xing et al., 2021), and the accumulation of autophagosomes in cells with extra centrosomes (Denu et al., 2020). Overall, this evidence suggests that cells with extra centrosomes could be important for cell-cell communication within the TME and stimulate the invasion of surrounding cancer cells during disease progression.

It is unclear, however, if tetraploid cells with extra centrosomes acquired via WGD also show a pro-invasive secretory phenotype associated with oxidative stress. Oxidative stress in MCF10A cells with extra centrosomes resulted from an increase in ROS production triggered by the stabilization of p53 (Arnandis et al., 2018). This may not occur in cancers that inactivate p53, a common event that precedes WGD during cancer evolution (Bielski et al., 2018), although there are other biological alterations associated with tetraploidy and aneuploidy that can lead to metabolic and oxidative stress (Biczowa et al., 1968; Anatskaya and Vinogradov, 2007; Li et al., 2010; Stingele et al., 2012; Shaukat et al., 2015; Newman et al., 2019). By inducing DNA damage and cell cycle delays, oxidative stress can lead to not only numerical, but also structural centrosome aberrations, which were also shown to contribute to cellular invasion (Ganier et al., 2018a; Ganier et al., 2018b). These findings suggest it may be necessary for cells with extra centrosomes to have some form of compensatory mechanism(s) that do not allow ROS generation, DNA damage, and centrosome amplification to exceed tolerable levels or increase their ability to tolerate oxidative stress. WGD, for example, could be one mechanism that increases a cell’s ability to tolerate oxidative damage, but this could also dampen the pro-invasive secretory response associated with oxidative stress and centrosome amplification.

Several studies have reported an association between centrosome abnormalities and metastasis risk (Sato et al., 1999; Hsu et al., 2005; LoMastro and Holland, 2019). Therefore, it will be important to determine whether cell ploidy affects the levels and/or functional effects of centrosome amplification-induced oxidative stress and whether centrosome amplification also promotes cancer cell invasion *in vivo*. One study found that, even with normal centrosome numbers, tetraploid cells were more migratory and invasive *in vitro* compared to diploid cells, but the authors also observed that tetraploid and/or aneuploid cells with extra centrosomes were enriched at the invasive margins of colon adenocarcinomas (Wangsa et al., 2018). This could be the result of high rates of WGD at the invasive fronts of cancers compared to more central regions. It could also indicate that cancer cells with extra centrosomes have a selective advantage at the tumor periphery or preferentially migrate towards the tumor margins to enable further invasion. Overall, these findings demonstrate that cells with extra centrosomes can facilitate invasion via cell-autonomous and non-cell-autonomous mechanisms, but more work is needed to fully understand these processes and their contribution to cancer progression. Since cellular invasion through the basement membrane is a critical step for cancer cell dissemination from the primary tumor site (Chambers et al., 2002; Sahai, 2007), centrosome amplification may be an important player driving metastasis in human cancers.

## 5 Extra centrosomes are not required for tetraploid cells to propagate genomic instability

**FIGURE 3:**
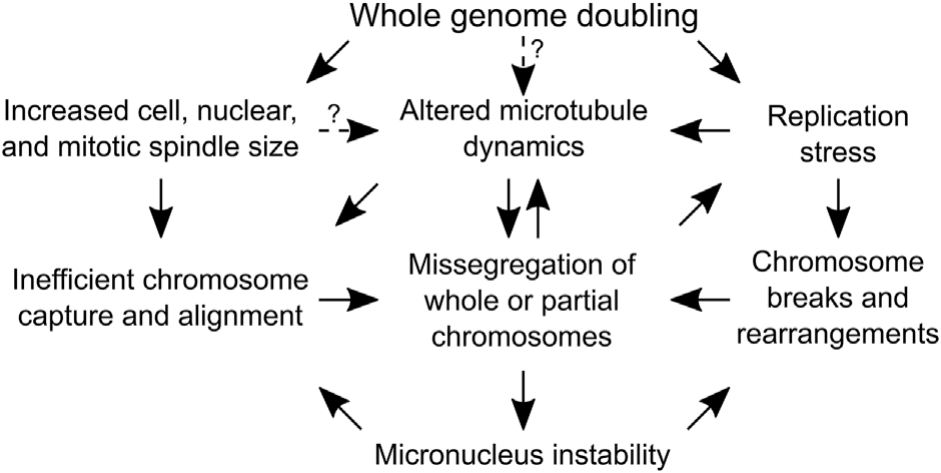
Causes of genome instability following whole genome doubling that do not depend on centrosome amplification. A flow chart depicting the mechanisms by which whole genome doubling can cause chromosome segregation errors. Arrows with solid lines are supported by experimental evidence, while arrows with dotted lines indicate the dependencies are speculative and require experimental validation.

**FIGURE 4:**
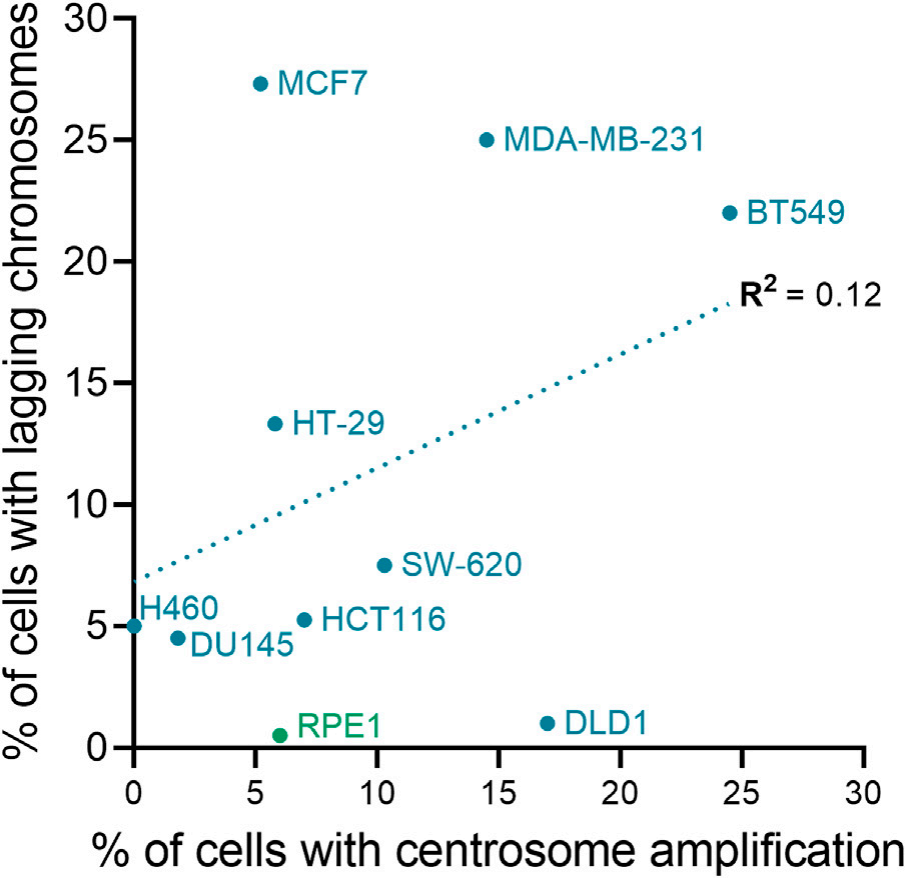
Centrosome amplification and chromosomal instability are not correlated in cell lines. The plot shows the relationship between chromosome missegregation (as determined by quantification of anaphase lagging chromosomes) and centrosome amplification in normal (green) and cancer (blue) cell lines. The linear fit and regression value (R^2^) are also shown in the graph. To calculate the fraction of ana-telophase cells with lagging chromosomes for each cell line, data from the Pellman (Ganem et al., 2009), Compton (Thompson and Compton, 2008), and Cimini (Silkworth et al., 2009; Nicholson and Cimini, 2013; Nicholson et al., 2015) labs were averaged (if applicable). Centrosome amplification data for the cell lines are from Marteil *et al.* (Marteil et al., 2018) and Baudoin *et al.* (Baudoin et al., 2020).

If retaining extra centrosomes leads to CIN and aneuploidy, then one potential evolutionary trade-off of restoring centrosome homeostasis would be less genomic diversity in tetraploid cell populations. This would be expected to slow the rate at which genomic alterations that increase fitness are acquired and hinder cancer evolution. However, there are a variety of mechanisms contributing to genomic instability in tetraploid cells that do not require supernumerary centrosomes (Figure 3). A majority, if not all, stable tetraploid clones have similar levels of centrosome amplification as the diploid cell lines from which they were derived (Ganem et al., 2009; Godinho et al., 2014; Kuznetsova et al., 2015; Wangsa et al., 2018; Bloomfield et al., 2021). Nevertheless, even with normal centrosome numbers, it is still common for tetraploid cells to missegregate chromosomes and accumulate more aneuploidy than diploid cells after only a few passages *in vitro* (Kuznetsova et al., 2015; Wangsa et al., 2018; Prasad et al., 2022). In fact, cancer cells can proliferate without centrosomes (Wong et al., 2015), there is evidence of CIN and whole chromosome aneuploidy in tetraploid acentriolar mouse embryos (Paim and FitzHarris, 2019), and centrosome defects, although common, were not correlated with WGD status in NCI-60 cell lines (Marteil et al., 2018). Supernumerary centrosomes also do not correlate with the degree of CIN in cancer cell lines (Figure 4). The colorectal cancer cell lines HCT-116 and HT-29 and breast cancer cell lines BT-549 and MCF-7 are some examples where CIN does not depend on centrosome defects. Despite having similar fractions of cells with abnormal centrosome numbers, the fraction of anaphase cells with lagging chromosomes in HT-29 is about twice that found in HCT-116. MCF-7 and BT-549 have similar CIN levels, even though centrosome amplification is almost two-fold higher in BT-549 than MCF-7 cells. Therefore, while possessing extra centrosomes does increase the risk of chromosome missegregation (Ganem et al., 2009), CIN in cancer cell lines appears to also be influenced by factors other than centrosome amplification, such as DNA content and the degree of aneuploidy (Nicholson and Cimini, 2013). It is important to note that this may not be true in non-transformed cell lines, like RPE-1. Indeed, tetraploid RPE-1 cells with extra centrosomes frequently missegregated chromosomes, while tetraploid RPE-1 cells without extra centrosomes displayed a chromosome missegregation rate similar to diploid RPE-1 cells (Ganem et al., 2009). Chromosome missegregation, however, may become more frequent in tetraploid RPE-1 cells as they accumulate aneuploidy or in specific tetraploid RPE-1 clonal populations (Kuznetsova et al., 2015; Harasymiw et al., 2019). Even if the chromosome missegregation rate does not change, a tetraploid genetic background is permissive to aneuploidy, allowing WGD+ normal and cancer cell lines to accumulate more chromosomal alterations over time than their parental WGD− cell lines (Dewhurst et al., 2014; Prasad et al., 2022).

Chromosome segregation defects in tetraploid cells, which maintain an active SAC, may result from ploidy-specific challenges in spindle assembly, chromosome alignment, and mitotic progression (Storchová et al., 2006; Kuznetsova et al., 2015; Viganó et al., 2018; Paim and FitzHarris, 2019; Bloomfield et al., 2021; Cohen-Sharir et al., 2021; Quinton et al., 2021). Tetraploid mammalian cells tend to take longer to complete mitosis than their diploid ancestors (Kuznetsova et al., 2015; Viganó et al., 2018; Paim and FitzHarris, 2019; Bloomfield et al., 2021; Quinton et al., 2021), possibly reflecting the challenges of capturing and aligning a larger number of chromosomes to satisfy and silence the SAC. The mitotic spindle is often larger in tetraploid relative to diploid cells due to the changes in cell size and ploidy, although studies in yeast and mammalian cells showed that spindle length does not always increase after WGD (Storchová et al., 2006; Bloomfield et al., 2021). We showed that cell and nuclear size as well as spindle geometry and composition did not consistently change in tetraploid clones relative to the diploid parental cells (Bloomfield et al., 2021). Relatively subtle differences in spindle architecture and cell size between tetraploid cells affected multiple mitotic events, including cell rounding, chromosome alignment, and mitotic timing (Bloomfield et al., 2021). A recent study found that large chromosomes near the periphery of the nucleus were more likely to end up near or behind a spindle pole during prometaphase and to be missegregated later in mitosis compared to chromosomes more centrally located in the nucleus (Klaasen et al., 2022). Since chromosome alignment efficiency also decreased as nuclear volume increased in tetraploid cells (Bloomfield et al., 2021), one consequence of a larger nucleus (and hence an expanded nuclear periphery) after WGD could be inefficient capture of peripheral chromosomes and an increased chance of chromosome missegregation in tetraploid cells. Along with increased aneuploidy tolerance, this could explain why gains and losses of larger chromosomes are more common in WGD+ compared to WGD− cancers (Prasad et al., 2022).

Recent findings also indicate that tetraploid (and aneuploid) cells are more sensitive than diploid cells to the depletion of specific mitotic proteins, such as KIF18A (Cohen-Sharir et al., 2021; Marquis et al., 2021; Quinton et al., 2021). The dependency of tetraploid cells on KIF18A, which suppresses microtubule growth to control chromosome oscillations at the metaphase plate (Stumpff et al., 2008; Stumpff et al., 2012), implies the possibility of ploidy-specific differences in microtubule dynamics. Depleting KIF18A led to centrosome fragmentation and spindle multipolarity that depended on dynamic microtubules in chromosomally unstable cancer cell lines, all of which have undergone WGD (Quinton et al., 2021), but not in stable near-diploid cell lines (Marquis et al., 2021). Similar to findings in WGD+ chromosomally unstable cancer cell lines (Bakhoum et al., 2009), kinetochore-microtubules attachments were hyper-stable and disrupted error correction in tetraploid mouse embryos (Paim and FitzHarris, 2019). This study also observed that aligned chromosomes were ejected from the metaphase plate and took several minutes to realign in tetraploid mouse cells, perhaps due to inefficient microtubule turnover (Paim and FitzHarris, 2019). KIF18A depletion led to a widening of the metaphase plate and oscillatory chromosome movements as well as high levels of chromosome missegregation in tetraploid, but not diploid, normal and cancer cells (Quinton et al., 2021), suggesting that high KIF18A levels might be required to temper microtubule dynamics in WGD+ cells.

Why would tetraploid cells display altered microtubule dynamics compared to diploid cells? One interesting possibility is that microtubule dynamics may need to change with cell size following WGD to maintain the time scales of cellular processes, which was observed during *C. elegans* embryogenesis (Lacroix et al., 2018). Although cell size was not reported, one study showed that microtubule polymerization rates were lower in two WGD+ clones compared to the near-diploid parental HCT-116 colorectal cancer cells (Cohen-Sharir et al., 2021). It is important to note that the HCT-116 WGD+ clones did not display higher KIF18 expression compared to diploid HCT-116 cells (Cohen-Sharir et al., 2021), whereas tetraploid MCF10A cells and many WGD+ human cancers expressed higher levels of KIF18A compared to near-diploid cells and WGD− cancers (Quinton et al., 2021). In this latter study, however, microtubule dynamics were not measured in the diploid and tetraploid MCF10A cells. Therefore, while there is evidence that microtubule dynamics and kinetochore-microtubule stability are altered in WGD+ cells, the underlying causes may vary. It is possible that microtubule dynamics in some WGD+ cells depend on cellular factors and molecular regulators other than KIF18A. For instance, an increase in microtubule plus-end assembly rates in aneuploid compared to near-diploid colorectal cancer cell lines led to spindle geometry defects, hyper-stable kinetochore attachments, and chromosome missegregation (Ertych et al., 2014). Changes in ploidy may also affect the mechanical properties of the centromere, which can impair the detection and destabilization of erroneous kinetochore-microtubule attachments (Harasymiw et al., 2019). Importantly, microtubule turnover is a requirement for the correction of kinetochore-microtubule attachment errors (Bakhoum et al., 2009; Bakhoum and Compton, 2012), so abnormal microtubule dynamics in WGD+ cells could lead to high rates of chromosome missegregation. It will be important for future studies to examine microtubule dynamics in tetraploid clones derived from multiple cell lines and to determine if microtubule dynamics change as WGD+ cells evolve.

Tetraploid cells also experience high levels of replication stress that can promote genomic instability (Pedersen et al., 2016; Wangsa et al., 2018), even during the subsequent S phase after WGD (Gemble et al., 2022). Replication stress can lead to DNA damage, chromosomal rearrangements, and chromosome missegregation (Pedersen et al., 2016; Wangsa et al., 2018; Wilhelm et al., 2019; Bernhard et al., 2022; Gemble et al., 2022). Following cytokinesis failure, U2-OS cancer cells displayed decreased DNA replication rates, a hallmark of replication stress, resulting in DNA damage and double strand breaks (Pedersen et al., 2016). A recent study found that tetraploid cells entered the first S phase after WGD with an insufficient level of key DNA replication factors and experienced high levels of DNA damage, chromosome over-duplication, and under- or over-replication of large genomic regions, which could be rescued by extending G1 duration (Gemble et al., 2022). This showed that replication stress can cause substantial genomic alterations even before tetraploid cells complete a full cell cycle following WGD, but it is unclear if such cells would remain viable or if G1 duration is also critically short following WGD *in vivo*. It is important to note that tetraploid cells will still have extra centrosomes immediately after WGD, but there is no evidence that having more centrosomes would directly lead to the replication defects observed in WGD+ cells. Tetraploid cells may also suffer from replication stress beyond the first cell cycle. Stable tetraploid clones derived from normal and cancer cell lines had higher baseline levels of replication stress and were more sensitive to inhibitors of DNA damage response proteins compared to the parental diploid cells (Wangsa et al., 2018). Due to the high levels of replication stress, tetraploid cells may also decrease the expression of specific proteins to weaken DNA damage response signaling and promote cell cycle progression (Bernhard et al., 2022). Moreover, mild replication stress and DNA damage were shown to cause chromosome missegregation by stabilizing microtubules and impairing the correction of kinetochore-microtubule attachment errors (Bakhoum et al., 2014; Wilhelm et al., 2019). These observations suggest that ongoing replication stress may contribute to the altered microtubule dynamics and CIN observed in tetraploid and aneuploid cancer cell lines.

Taken together, these findings demonstrate that tetraploid cells experience genomic instability from multiple sources, many of which do not depend on supernumerary centrosomes. For instance, tetraploid cells with normal centrosome numbers can experience replication stress and chromosome segregation defects that lead to gene mutations and aneuploidy. Moreover, since tetraploid cells appear to tolerate aneuploidy better than diploid cells, even low levels of chromosome missegregation will lead to the accumulation of aneuploidy and allow tetraploid cells to explore more genomic and karyotypic space than diploid cells (Prasad et al., 2022). WGD+ cells tend to favor chromosome loss and, unlike WGD− cells, may eventually converge on near-triploid karyotypes if kept in culture for extended periods (Dewhurst et al., 2014; Laughney et al., 2015), similar to what is observed in WGD+ cancers (Carter et al., 2012; Zack et al., 2013; Dewhurst et al., 2014; Bielski et al., 2018; Taylor et al., 2018; Prasad et al., 2022).

## 6 Is there a link between whole genome doubling and centrosome amplification in human cancers?

WGD is a frequent event in cancer that has important clinical implications (Bielski et al., 2018), but there is still much that we do not understand about how cancers develop after tetraploidization. It is thought that tetraploidy in cancer results primarily from non-programmed, or erroneous, WGD events (Davoli and de Lange, 2011). WGD also occurs in healthy human tissues, including the heart and liver, as a part of development or in response to aging, stress, or injury (Davoli and de Lange, 2011; Schoenfelder and Fox, 2015), but programmed polyploidy is generally associated with terminal differentiation (Lee et al., 2009) and does not pose a threat to tissue homeostasis. Hepatocytes represent an exception to this rule, as they can, under certain conditions, proliferate after WGD and accumulate aneuploidy (Duncan et al., 2010; Duncan et al., 2012; Matsumoto et al., 2020). Most evidence suggests that in this context, tetraploidy plays a tumor suppressive role in the liver by buffering the effects of harmful mutations (Zhang et al., 2018a; Zhang et al., 2018b; Lin et al., 2020; Wang et al., 2021). Notably, around 30% of liver hepatocellular carcinomas display evidence of WGD (Quinton et al., 2021), which is similar to other tissues that are predominantly diploid (Zack et al., 2013; Bielski et al., 2018), suggesting that the physiological tetraploidy in the liver does not predispose this organ to tumorigenesis. Interestingly, the incidence of  WGD varies across different cancer types, ranging from less than 10% in pancreatic and thyroid carcinomas to over 50% in breast, lung, and ovarian carcinomas (Quinton et al., 2021). These observations suggest that cell type- and/or tissue-specific factors other than the physiological levels of tetraploidy may determine whether WGD prevents or promotes cancer. For instance, nutrient availability may be an important factor for the expansion of tetraploid cell populations in different tissues (Kimmel et al., 2020; Andor et al., 2022). Experimental findings have demonstrated that WGD often triggers a p53-mediated cell cycle arrest in non-transformed cells (Andreassen et al., 2001; Kuffer et al., 2013), which can be overcome by some tetraploid cells if the p53, Rb, and/or Hippo pathways are disrupted or if cyclin D and/or E are over-expressed (Andreassen et al., 2001; Fujiwara et al., 2005; Castedo et al., 2006; Ganem et al., 2014; Crockford et al., 2017; Zeng et al., 2023). Tetraploid cells can also be targeted and cleared by immune cells (Senovilla et al., 2012), similar to aneuploid cells (Santaguida et al., 2017). Therefore, despite the prevalence of WGD in cancer, there are several mechanisms that appear to limit the oncogenic potential and evolutionary success of newly formed tetraploid cells in healthy tissues.

One open question is whether maintaining or losing the extra centrosomes would be more advantageous for the evolution of newly formed tetraploid cells. Given the prevalence of numerical centrosome abnormalities in cancer (Chan, 2011) and their effect on mitotic fidelity (Ganem et al., 2009; Silkworth et al., 2009), it has been proposed that the extra centrosomes acquired during WGD play a key role in cancer evolution by promoting chromosomal instability and aneuploidy in tetraploid cells (Storchova and Pellman, 2004; Ganem et al., 2007). Most experimental evidence for this theory, however, is circumstantial. In their seminal paper, Fujiwara *et al.* found that tetraploid, but not diploid, mouse mammary epithelial cells were tumorigenic in mice and more than half of the cells recovered from tumors had extra centrosomes (Fujiwara et al., 2005). While this study effectively showed tetraploidy is sufficient to drive tumor formation *in vivo*, it did not fully elucidate the role of centrosome amplification in this process. For one, centrosome amplification was determined based on the number of pericentrin foci, which do not always contain centrioles (Pihan et al., 1998) and are not the most accurate readout of supernumerary centrosomes. Additionally, it is unclear what fraction of cells with supernumerary centrosomes in the mouse tumors were proliferative (i.e., actively contributing to tumor expansion), were arrested, never proliferated after WGD (tetraploid and binucleate), or underwent a second WGD event (octoploid and possibly bi- or poly-nucleated). Therefore, thorough quantification of ploidy and proliferation status of cancer cells with centrosome amplification is necessary to better understand the role of supernumerary centrosomes in tetraploidy-induced tumorigenesis.

Is there any evidence that WGD and centrosome amplification are linked in human tumors? One study found that WGD and CA20 score, which is based on the expression of genes associated with centrosome amplification (Ogden et al., 2017), were correlated in samples from The Cancer Genome Atlas (TCGA) (de Almeida et al., 2019), but this does not show what fraction of cells have supernumerary centrosomes and how centrosome numbers evolved after WGD in these cancers. It is inherently difficult to examine centrosome number evolution in clinical samples, since longitudinal sampling is not always feasible and WGD may occur long before cancer detection. A rare example where clinical specimens are collected at multiple stages of cancer progression is in patients with Barrett’s esophagus (BE), a condition that increases the risk of esophageal adenocarcinoma (EAC) (Cameron et al., 1985; Hameeteman et al., 1989). In this human cancer model, loss of p53 and tetraploidy are observed in precancerous lesions, which leads to aneuploidy and the emergence of neoplastic cell populations during progression to EAC (Galipeau et al., 1996). Therefore, this is an ideal system to study centrosome number evolution in WGD+ human tumors. One study that analyzed patient samples using immunohistochemical staining reported that pericentriolar defects decreased in patient samples during neoplastic progression from BE to EAC (Segat et al., 2010). More recently, centriole numbers were analyzed over time in patient samples using immunofluorescence. The authors found that the fraction of cells with supernumerary centrosomes in patient samples, which often remained below 10%, increased during the transition from metaplasia to dysplasia, decreased upon neoplastic progression to adenocarcinoma, and then increased in lymph node metastases (Lopes et al., 2018). Corresponding genomic analyses were not performed, so it is unclear what fraction of the cell population analyzed was diploid, tetraploid, or aneuploid at each stage and whether it correlated with the fraction of cells with extra centrosomes. Centrosome amplification in precancerous lesions is presumably linked to WGD, which occurs in BE before EAC development (Galipeau et al., 1996). It is tempting to speculate that the decrease in centrosome amplification during the transition from dysplasia to adenocarcinoma may reflect the loss of extra centrosomes in newly formed tetraploid cells as they proliferate and transform into aneuploid neoplastic cells. In cell lines derived from metaplastic BE samples, near-tetraploid cells with normal and abnormal centrosome numbers were observed (Lopes et al., 2018), but these cells could have lost centrosomes during *in vitro* propagation. Cancer cells with supernumerary centrosomes, which may provide a selective advantage in the metastatic process by promoting cellular invasion (Godinho et al., 2014; Arnandis et al., 2018), found in lymph nodes metastases may have either retained the extra centrosomes acquired during WGD or subsequently acquired additional centrosomes after initial centrosome loss. Some studies have also analyzed centrosome number defects in patients with ovarian cancer, which has a high rate of  WGD (Zack et al., 2013; Bielski et al., 2018). Although ploidy or WGD status was not reported, centrosome amplification was not widespread in clinical samples and was only present in small populations of epithelial ovarian cancer cells (Morretton et al., 2022). A recent preprint that examined centrosome numbers in clinical samples from patients with high grade serous ovarian cancer found that centrosome amplification was not correlated with tumor ploidy and was driven by centriole overduplication rather than WGD (Sauer et al., 2022). Taken together, these findings suggest that esophageal and ovarian cancer cells may lose extra centrosomes after WGD *in vivo*, but further characterization of centrosome number dynamics in tetraploid and aneuploid cells in other tumor types where WGD is common will be essential to understand the mechanisms that lead to centrosome amplification in human cancers.

Centrosome amplification has been reported in many cancers (Chan, 2011), but a large fraction of these studies have relied on histological staining of PCM markers in tumor specimens that can be difficult to interpret and prone to inaccuracies (Zyss and Gergely, 2009). Overall, there is still controversy regarding the role of centrosome amplification in tumorigenesis. Extra centrosomes are thought to promote cancer formation by inducing genomic instability, but it is possible that too much centrosome amplification could inhibit cancer formation. High levels of centrosome amplification, in turn, may lead to high levels of aneuploidy and CIN that are not sustainable for evolving cell populations. There is evidence that high levels of aneuploidy inhibit tumor formation in mice (Weaver et al., 2007). This is also true in mouse models where aneuploidy is a consequence of increasing centrosome numbers (Marthiens et al., 2013; Kulukian et al., 2015; Vitre et al., 2015). In the brain and epidermis of mice, centrosome amplification-induced aneuploidy led to p53-mediated cell death and did not lead to tumor formation (Marthiens et al., 2013; Kulukian et al., 2015). Even in a model of chronic Plk4 over-expression, some tissues, such as the lung and kidney, were shown to not tolerate centrosome amplification and reduce the number of cells with extra centrosomes (Vitre et al., 2015). The same study also found that p53 prevents the proliferation of cells with supernumerary centrosomes, and centrosome amplification did not affect tumor incidence in p53-heterozygous or p53-null mice that are predisposed to developing tumors in a variety of tissues (Vitre et al., 2015). These findings suggest that an excessive amount of centrosome amplification and aneuploidy can suppress tumorigenesis in mice.

Several other studies, however, showed that both chronic and transient centrosome amplification can promote tumorigenesis in p53-deficient mice that are more permissive to aneuploidy than mice with wild-type p53 (Coelho et al., 2015; Serçin et al., 2016; Shoshani et al., 2021). There is also evidence that low levels of chronic or transient centrosome amplification can lead to tumor formation in mice with functional p53, but the levels of p53 transcript varied and the expression of p53 targets was low in the resulting lymphomas (Levine et al., 2017). These findings suggests that a low-to-moderate level of centrosome amplification can cause tumorigenesis in mice, perhaps by limiting the resulting aneuploidy and CIN to optimal levels like what has been observed in other experimental systems (Weaver et al., 2007). Additionally, these studies provide further support that the proliferation and survival of cells with abnormal centrosome and chromosome numbers during cancer evolution are readily improved by weakening or inactivating the p53 pathway. Nevertheless, loss of p53 function is not sufficient for centrosome amplification, and p53-deficient tetraploid cells do not maintain high levels of centrosome amplification *in vitro* (Ganem et al., 2009; Baudoin et al., 2020; Galofré et al., 2020; Sala et al., 2020). Although some cell lines are able to maintain high levels of centrosome amplification (Marteil et al., 2018; Adams et al., 2021), the tendency for newly formed tetraploid cells to reduce centrosome number suggests that centrosome amplification impairs cell fitness and would be selected against during the evolution of a cell population (Dias Louro et al., 2021). These findings suggest that the loss of extra centrosomes in newly formed tetraploid cells may be beneficial for cancer progression after WGD by preventing excessive and detrimental levels of aneuploidy and CIN.

## 7 Concluding Remarks

It is generally assumed that WGD promotes tumorigenesis due to the ability of the extra centrosomes acquired during WGD to induce CIN. However, as summarized in this review, a closer look at the available evidence reveals that this may not or not always be the case. It seems probable that centrosome numbers do not remain static and instead undergo dynamic evolution—much like chromosome numbers—to find a stable equilibrium following WGD in cancers. One possibility is that instead of maintaining high levels of centrosome amplification following WGD, it may be beneficial for at least some newly formed tetraploid cells to lose their extra centrosomes in the early stages of cancer development. This would create a presumably more stable  WGD+ cancer cell population with normal centrosome numbers, while other WGD+ cancer cells could retain extra centrosomes or acquire additional centrosomes. There are several factors that can promote centrosome amplification in cancer cells. It will be important to understand if WGD and/or aneuploidy make cancer cells prone to centrosome duplication errors in response to cellular or environmental stress. The link between DNA damage and centrosome amplification could be especially relevant to WGD+ cells, which are more susceptible to replication stress and DNA damage than WGD− cells (Wangsa et al., 2018; Gemble et al., 2022). Nevertheless, even in cases where cellular errors or environmental stress are associated with abnormal centrosomal or mitotic figures, it is not always clear whether this is caused by centriole splitting, overduplication, *de novo* assembly, or WGD events. Delineating the molecular mechanism(s) underlying centrosome amplification observed in cell lines and clinical samples has also proven challenging due to overlap in regulatory pathways governing the DDR, cell cycle progression, and centrosome duplication and the inherent limitations of *in vivo* analysis. To comprehensively characterize centrosome amplification in WGD+ and WGD− cancers, it will be important to analyze both ploidy and centriole numbers in single cells, differentiate between proliferative and arrested cancer cells with supernumerary centrosomes, and, if possible, determine how centrosomes are clustered in mitotic cells and/or distributed to the progeny of cancer cells dividing with extra centrosomes. This will provide further insights into how centrosome and chromosome numbers co-evolve after WGD and contribute to cancer progression.
